# The Health Reports

**Published:** 1880-08

**Authors:** 


### THE HEALTH REPORTS.

In our last number we indulged in kindly criticism of the weekly reports issued by the Board of Health, and pointed out defects in the method adopted to furnish information to the public and the profession in regard to the sanitary condition of the city. We disclaimed any intention to impugn the motives or to embarrass the efforts of the Health Physician, Dr. Briggs, whose energy in the special work committed to his charge deserves all praise. Acting upon the suggestions made with a view especially to secure data concerning the influence of the Hamburg Canal as a factor in the causation of malarial and zymotic diseases, the Board of Health have issued a circular, accompanied with blanks, in which the active co-operation of the profession is desired in sending weekly a record of all cases, occurring within the city limits for the space of two months, from July 15 to September 15. Certain cases are wisely excepted.

We cannot enter into a discussion of the plan adopted by the health authorities, nor do we desire to point out its defects. We take the position that, as conservators of the public health, it is the duty of the medical profession to second every effort made by the legally constituted authorities to improve the sanitary condition of the city. In the present case information is desired, which the profession can, with a small expenditure of time and effort, furnish. Its ulterior object is to add a conclusive argument to the many already adduced, against an evil, the prolonged continuance of which will endanger the lives of our people. Now, whether this is the only or the best manner to secure the end desired to be attained by the Board of Health, we do not stop to question, but as it is sanctioned by lawful authority, it is the plain duty of physicians to give it a willing endorsement. Objections may be raised in obscure and irresponsible sources, but every enlightened physician who labors for the public weal, rather than for private and individual ends, should second the praiseworthy attempt to gather valuable statistics as to the influence of local causes over the type and character of morbid action, especially if by rendering such aid, the public for which the profession stand as sponsors in sanitary matters, will be benefited thereby.

